# Changes in accommodation and behavioural performance with a contact lens for myopia management: A comparison between a dual‐focus and a single‐vision soft contact lens

**DOI:** 10.1111/opo.12978

**Published:** 2022-03-19

**Authors:** Beatriz Redondo, Jesús Vera, Rubén Molina, Tomás Galán, Pedro Machado, Raimundo Jiménez

**Affiliations:** ^1^ CLARO (Clinical and Laboratory Applications of Research in Optometry) Research Group, Department of Optics, Faculty of Sciences University of Granada Granada Spain

**Keywords:** accommodation, behavioural performance, dual‐focus soft contact lenses, myopia management

## Abstract

**Introduction:**

Dual‐focus soft contact lenses for myopia management have demonstrated to be an effective strategy to reduce myopia progression. However, this optical design has been shown to alter visual quality and accommodative function. The aim of this study was to examine the accommodative and behavioural performance during the execution of a psychomotor vigilance task (PVT) while wearing dual‐focus and single‐vision soft contact lenses.

**Methods:**

The steady‐state accommodative response was recorded with the WAM‐5500 binocular open‐field autorefractor during the execution of a 10‐min PVT at 50 cm either with the dual‐focus (MiSight 1‐day) or single‐vision (Proclear 1‐day) soft contact lenses, using a sample of 23 healthy young adults. Each experimental session was performed on two different days in a counterbalanced order.

**Results:**

A greater lag of accommodation, variability of accommodation and reaction time was found while wearing dual‐focus in comparison with single‐vision soft contact lenses (mean differences during the 10‐min PVT were 0.58 ± 0.81 D, *p* < 0.001; 0.31 ± 0.17 D, *p* < 0.001 and 15.22 ± 20.93 ms, *p* = 0.002, respectively). Also, a time‐on‐task effect was found for the variability of accommodation and reaction time (*p* = 0.001 and *p* < 0.001, respectively), observing higher values over time. However, the lag of accommodation did not change significantly as a function of time‐on‐task (*p* = 0.33).

**Conclusion:**

Dual‐focus soft contact lens wear influences the steady‐state accommodative response and behavioural performance during the execution of a visual vigilance task in the short‐term. Eye care practitioners should be aware of these effects when prescribing these lenses for myopia management, and provide specific recommendations according to the individual visual needs.

**Key points**

- Dual‐focus soft contact lenses increase the lag and variability of accommodation.
- Visual performance is negatively affected by dual‐focus soft contact lenses.
- Dual‐focus soft contact lenses should be prescribed according to individual visual needs.

## INTRODUCTION

Myopia has become a major public health issue due to the substantial increase in prevalence in the last decades, and its association with the onset of different ocular pathologies (cataract, chorioretinal atrophy, macular hole, myopic foveoschisis, optic nerve head changes, etc.).^1^ Myopia, and especially high degrees of myopia (>6D), increases the chances of irreversible blindness in the long‐term.^2^, ^3^, ^4^ Traditional optical correction of the underlying refractive error does not prevent myopia progression, and consequently the appearance of potential ocular pathologies linked to myopia.^5^ Because of this, the research community has focused on testing the efficacy of a range of optical strategies to prevent myopia progression.^6^, ^7^, ^8^, ^9^, ^10^ In this regard, different multifocal soft contact lens designs with myopia management features have raised great interest. These contact lenses have been designed to reduce hyperopic and induce myopic defocus, by manipulating the optical power in the lens periphery (i.e., spherical areas of positive power,^11^ multiple concentric treatment zones^12^ and induction of spherical aberration),^13^ which is considered to play a key role in myopia development and progression.^14^

Although this strategy has shown favourable effects for myopia management,^15^ there is scientific evidence showing that vision quality is degraded while wearing these lenses.^16^, ^17^, ^18^, ^19^ For example, wearing multifocal soft contact lenses has been associated with an increased accommodative lag, which may lead to reduced image quality during near work.^20^, ^21^, ^22^ Also, contrast sensitivity, light disturbance, higher order aberrations and visual comfort scores have been shown to be negatively affected by multifocal soft contact lenses used for slowing myopia progression, although no changes in visual acuity and stereopsis have been observed.^16^, ^17^, ^23^, ^24^, ^25^, ^26^

Many everyday activities require precise visual capacities (e.g., reading, driving, piloting, air traffic control, etc.), and thus, optimal task performance depends on preserved visual functioning. Therefore, retinal defocus exceeding the depth of focus of the eye has been shown to compromise reading performance and digit recognition speed when the defocus is greater than 2 D and between 1.0–1.5 D, respectively.^27^, ^28^ In addition, Poltavski et al. artificially induced accommodative‐vergence stress with −2.0 D lenses, and found that a higher accommodative lag was associated with worse performance on a neuropsychological task of sustained attention (Conners' Continuous Performance Test).^29^

In view of this previously reported scientific evidence, it seems plausible to expect that dual‐focus soft contact lens wear may have a negative effect on visual function and behavioural performance (visual reaction time) during the execution of cognitively demanding visual tasks at near. Specifically, a visual psychomotor vigilance task (PVT), which is a sustained attention, reaction‐timed task that measures the speed of responding to a visual stimulus presented at random inter‐stimulus intervals. To date, there are no studies that have tested this hypothesis, and therefore this study was designed to fill this research gap. The main objective was to compare accommodative and behavioural performance during the execution of a visual vigilance task while using dual‐focus (MiSight 1‐day) and single‐vision (Proclear 1‐day) soft contact lenses in a sample of healthy young adults. To do so, we recorded the steady‐state accommodative response with a binocular open‐field autorefractor while participants performed a 10‐min visual PVT at 50 cm with both types of soft contact lenses (dual‐focus vs. single‐vision). Our hypothesis is that dual‐focus lens wear will impair accommodation, as has been shown for a range of visual skills,^16^, ^17^, ^20^, ^21^, ^22^, ^23^, ^24^, ^25^, ^26^ and this alteration of visual functioning could cause reduced behavioural performance during the vigilance task.

## METHODS

### Participants

We performed an a‐priori sample size calculation, assuming an effect size of 0.25, power of 0.80 and alpha of 0.05, which projected a required sample size of 22 subjects for this experimental design.^30^ As a result, 23 university students (mean age ± standard deviation: 20.46 ± 2.02 years) were recruited to participate in the study. Participants were screened according to the following inclusion criteria: (i) free of any systemic or ocular disease; (ii) no history of strabismus, amblyopia or refractive surgery; (iii) soft contact lens wearers with myopia <−0.50 D and astigmatism <0.75 D; (iv) have normal or corrected‐to‐normal vision (visual acuity of ≤0.00 logMAR in each eye); (iv) accommodative lag <1.55 D at 20 cm, when measured by three static measurements with the WAM‐5500 binocular open‐field autorefractor, which is considered to be within the normal range;^31^ (v) low visual discomfort symptomatology based on the Conlon visual discomfort survey (≤24)^32^ and (vi) a score <4 on the Stanford Sleepiness Scale (SSS), which provides a global measure of sleepiness.^33^ All participants were asked to abstain from alcohol and caffeine‐based drinks for 24 and 12 h before each experimental session, respectively, and to sleep at least 7 h during the nights prior to testing. The experimental protocol followed the guidelines of the Declaration of Helsinki and was approved by the University of Granada Institutional Review Board (IRB approval: 1786/CEIH/2020).

### Accommodative response

**FIGURE 1:**
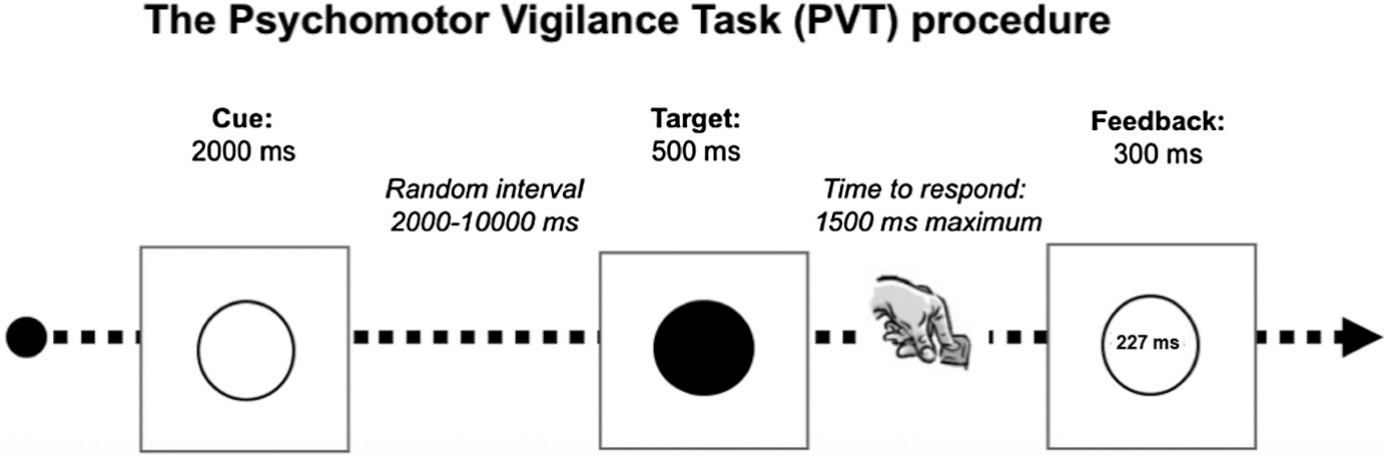
Schematic illustration of the visual psychomotor vigilance task used in this study

The accommodative lag and variability were measured with the clinically validated WAM‐5500 binocular open‐field autorefractor (Grand Seiko, grand‐seiko.com) in hi‐speed mode. This instrument has been demonstrated to measure the refractive state accurately for different accommodative demands in eyes fitted with multi‐zone bifocal contact lenses.^34^ First, we obtained monocular static refractive measures for both corrected eyes while participants viewed a 5 m stationary target (residual refractive error). Following this measurement, the 10‐min PVT commenced. The accommodative response was measured continuously (without breaks) under binocular viewing conditions, although refractive state measurements were obtained from the dominant eye, as determined by the sighting method.^35^ Participants were instructed to maintain focus on a high‐contrast visual stimulus from the PVT on a 15.6″ LCD screen placed at 50 cm from the observer (see Psychomotor vigilance task and Figure 1 for a detailed explanation of this task). During dynamic recording, an experienced examiner verified that the autorefractor remained aligned with the fixation target to ensure on‐axis measurements by maintaining the alignment target within the pupil centre in the LCD monitor. The room illumination was kept constant during the experiment (212 ± 9 lux as measured in the corneal plane; T‐10, Konica Minolta, konicaminolta.com).

For data analysis, we identified and removed any data points ±3 standard deviations from the mean spherical refraction value, which could be caused by blinking or recording errors.^36^ Accommodative lag was calculated by subtracting the accommodative response from the target distance (2D) after considering the residual refractive error.^29^ The variability of accommodation referred to the standard deviation of the dynamic accommodative recording.

### Psychomotor vigilance task (PVT)

The PVT is a sustained attention reaction‐timed task that measures the speed with which subjects respond to a visual stimulus presented at random inter‐stimulus intervals (Figure 1).^37^ Here, we included a modified version of the PVT with a duration of 10 min that used a 15.6″ LCD laptop personal computer and E‐Prime software (Psychology Software Tools, pstnet.com) for stimulus presentation and response collection. The visual stimulus was presented at 50 cm and consisted of an empty, high‐contrast black circle (viewing angle of 6.73°) on a white background. At a random time interval (between 2000 and 10,000 ms), the circle became filled in at once in black. When participants detected the filled circle, they were instructed to press the space bar on the keyboard as fast as they could with their dominant hand. Reaction time was defined as the length of time between the stimulus presentation and the participant's response. The filled circle was presented for 500 ms and the maximum time to respond was 1500 ms. A reaction time visual feedback message was presented after each response, except in case of an anticipated response (“wait for the target”) or if no response was made within 1000 ms after target offset (“you did not answer”). After the feedback message, the next trial commenced.

### Procedure

**FIGURE 2:**
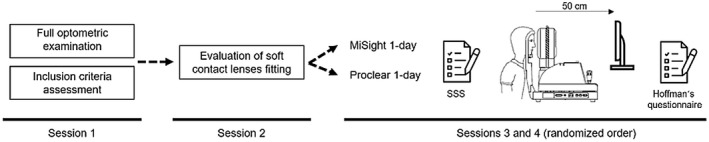
A graphical overview of the procedure followed in the current study. Abbreviation: SSS, Stanford Sleepiness Scale

Participants attended the laboratory on four different days, and the procedure followed is depicted in Figure 2. In the first session, participants received a full optometric examination to verify that they met the inclusion criteria and to determine the required optical correction. The average contact lens power was −2.92 ± 1.61 D and −3.11 ± 1.43 D for the right and left eyes, respectively (anisometropia ranged from 0 to −1.5 D). For each optical design, namely dual‐focus (MiSight 1‐day; CooperVision, coopervision.com) and single‐vision (Proclear 1‐day; CooperVision, coopervision.com) soft contact lenses, both being hydrophilic contact lenses composed of omafilcon A material, the same contact lens power was prescribed. The MiSight contact lens design consists of a central correction zone surrounded by a series of concentric treatment zones of +2.0 D. The first session included an objective ocular refraction and keratometry using the WAM‐5500 autorefractor; monocular and binocular subjective refraction using an end point criterion of maximum plus consistent with best vision; assessment of the accommodative and binocular function following the recommendations of Scheiman and Wick^38^ and the identification of any ocular pathology by slit‐lamp and direct ophthalmoscopy. Then, both sets of soft contact lenses were ordered based on corneal measurements and the refractive error after compensation for the vertex distance. In the second session, we evaluated whether the contact lenses were appropriately centred, had adequate movement (which was accomplished in all cases) and an over refraction performed to ensure appropriate visual acuity (≤0.00 logMAR in each eye). The remaining two sessions constituted the main experimental trials. Each were identical except for the type of contact lens used, with the order of these sessions being counterbalanced. Upon arrival at the lab, participants completed the SSS, which consists of a 7‐point scale, ranging from 1 “very active, alert or awake” to 7 “very sleepy”.^23^ After this, the corresponding contact lens (MiSight or Proclear) was fitted. To accomplish the double‐blind procedure, neither the examiner nor participant were aware of the contact lens type, as the lenses were prepared by a third person. After wearing the lenses for 30 min to allow adaptation,^39^ participants sat in front of the computer in a dimly illuminated room, isolated from external noise and positioned their chin and forehead on the respective supports of the autorefractor. Prior to testing, they received instructions and practiced for 1 min with the PVT. At this point, the 10‐min PVT started, and accommodative function was monitored during the entire period. When the PVT ended, we assessed the subjective levels of visual fatigue and discomfort with a 5‐point Likert scale developed by Hoffman et al., which includes the following items: (1) How tired are your eyes?, (2) How clear is your vision?, (3) How tired and sore are your neck and back?, (4) How do your eyes feel?” and (5) How does your head feel? In order to assess the time‐on‐task effects while performing the 10‐min task, the lag and variability of accommodation and reaction time were divided into five blocks of 2 min each.^40^

### Experimental design and statistical analysis

The present study followed a double‐blind, balanced crossover design. The within‐participants factors were the contact lens (MiSight or Proclear) and time‐on‐task (five 2‐min blocks: block 1, block 2, block 3, block 4 and block 5). The dependent variables were the lag and variability of accommodation and behavioural performance (reaction time).

Prior to any statistical analysis, the normal distribution of the data (Shapiro–Wilk test) and the homogeneity of variances (Levene's test) were confirmed (*p* > 0.05). To check for possible differences in the level of alertness at the beginning of each experimental session, a *t*‐test for related samples was performed for the SSS. Also, a *t*‐test for related samples with the use of MiSight or Proclear contact lenses as the only within‐participants factor was carried out for the five questions in Hoffman's questionnaire. Three separate two‐way analyses of variance (ANOVAs) with the lens type and time‐on‐task as the within‐participants factors were performed to test the impact of the experimental manipulation on accommodative lag, variability of accommodation and behavioural performance. The magnitude of the differences was reported by the partial eta squared (ƞ_p_^2^) and Cohen's effect size (ES) for Fs and *t*‐tests, respectively. Statistical significance was set at an alpha level of 0.05, and *post‐hoc* tests were corrected with the Holm‐Bonferroni procedure. The JASP statistics package (version 0.13.1.0; jasp‐stats.org) was used for statistical analyses.

## RESULTS

Analysis of the SSS indicated that participants attended each experimental session with similar level of alertness/sleepiness (MiSight: 2.04 ± 0.75; Proclear: 2.00 ± 0.72; t_23_ = 0.25, *p* = 0.80). Note that a score of 2 in the SSS is defined as “Functioning at high levels, but not at peak; able to concentrate”. The subjective levels of visual fatigue and discomfort after performing the PVT revealed only statistically significant differences for the second item, which refers to the clarity of vision (t_23_ = 4.73, *p* < 0.001) (Table 1).

**TABLE 1:** Descriptive (mean ± standard deviation) and statistical (*p*‐value and Cohen's d) values of the reported symptoms of visual fatigue and discomfort after completing the 10‐min psychomotor vigilance task with the MiSight and Proclear contact lenses

	MiSight	Proclear	*p*‐Value	Cohen's d
1) How tired are your eyes? (0–4)	1.71 ± 0.86	1.38 ± 0.82	0.18	0.29
2) How clear is your vision? (0–4)	2.04 ± 0.81	0.83 ± 0.76	<0.001	0.97
3) How tired and sore are your neck and back? (0–4)	1.58 ± 0.93	1.38 ± 1.01	0.17	0.29
4) How do your eyes feel? (0–4)	1.54 ± 0.72	1.29 ± 0.62	0.11	0.34
5) How does your head feel? (0–4)	1.21 ± 0.72	1.08 ± 0.78	0.48	0.15

*Note:* The questionnaire of visual fatigue and discomfort was developed by Hoffman et al.^40^ The five items of this 5‐point Likert scale ranged from 0 (no symptoms) to 4 (severe symptoms).

**FIGURE 3:**
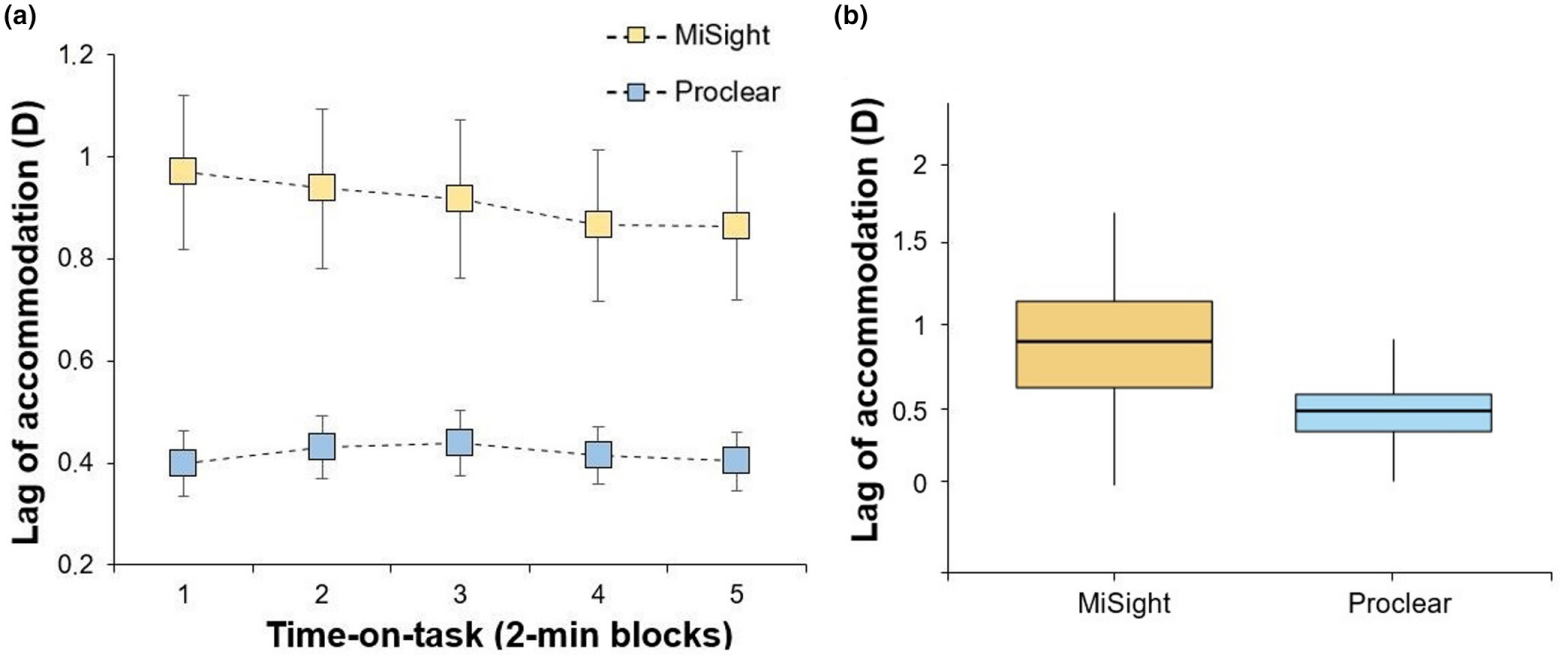
(a) Mean lag of accommodation during the five time‐on‐task blocks for the MiSight and Proclear lenses. Error bars indicate one standard error. (b) Boxplot showing the mean lag, with the whiskers extending 1.5 times the interquartile range from the 25th and 75th percentiles

**FIGURE 4:**
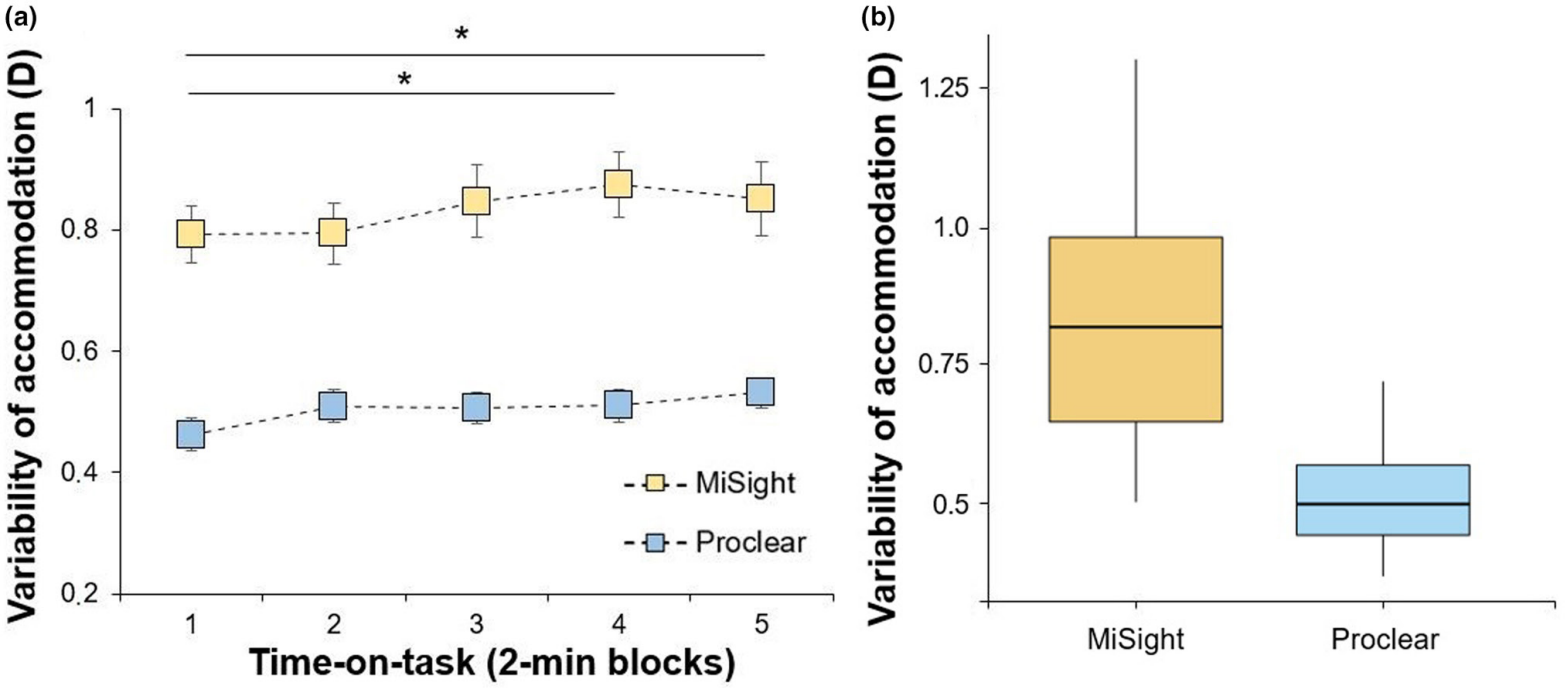
(a) Mean variability of accommodation during the five time‐on‐task blocks for the MiSight and Proclear lenses. Error bars indicate one standard error. (b) Boxplot showing the mean variability with the whiskers extending 1.5 times the interquartile range from the 25th and * denotes statistically significant differences between the different time blocks (corrected *p*‐value < 0.05)

Lens type produced statistically significant differences in the lag of accommodation (MiSight: 1.00 ± 0.85 D; Proclear: 0.42 ± 0.28 D; *F*_1,23_ = 14.91, *p* < 0.001, ƞ_p_^2^ = 0.39), but not for the time on task (*F*_4,92_ = 1.17, *p* = 0.33) nor the interaction lens type × time‐on‐task (*F*_4,92_ = 1.16, *p* = 0.33) (Figure 3). The variability of accommodation exhibited statistically significant differences for lens type (MiSight: 0.84 ± 0.22 D; Proclear: 0.52 ± 011 D; *F*_1,23_ = 81.51, *p* < 0.001, ƞ_p_^2^ = 0.78) and time‐on‐task (*F*_4,92_ = 4.84, *p* = 0.001, ƞ_p_^2^ = 0.17), but not for the interaction lens type × time‐on‐task (*F*_4,92_ = 1.74, *p* = 0.15) (Figure 4). *Post‐hoc* analyses for the point of measure are depicted in Figure 4a and indicated statistically significant differences when comparing block 1 with block 4 (corrected *p*‐value = 0.005, ES = 0.74) and block 5 (corrected *p*‐value = 0.005, ES = 0.73).

**FIGURE 5:**
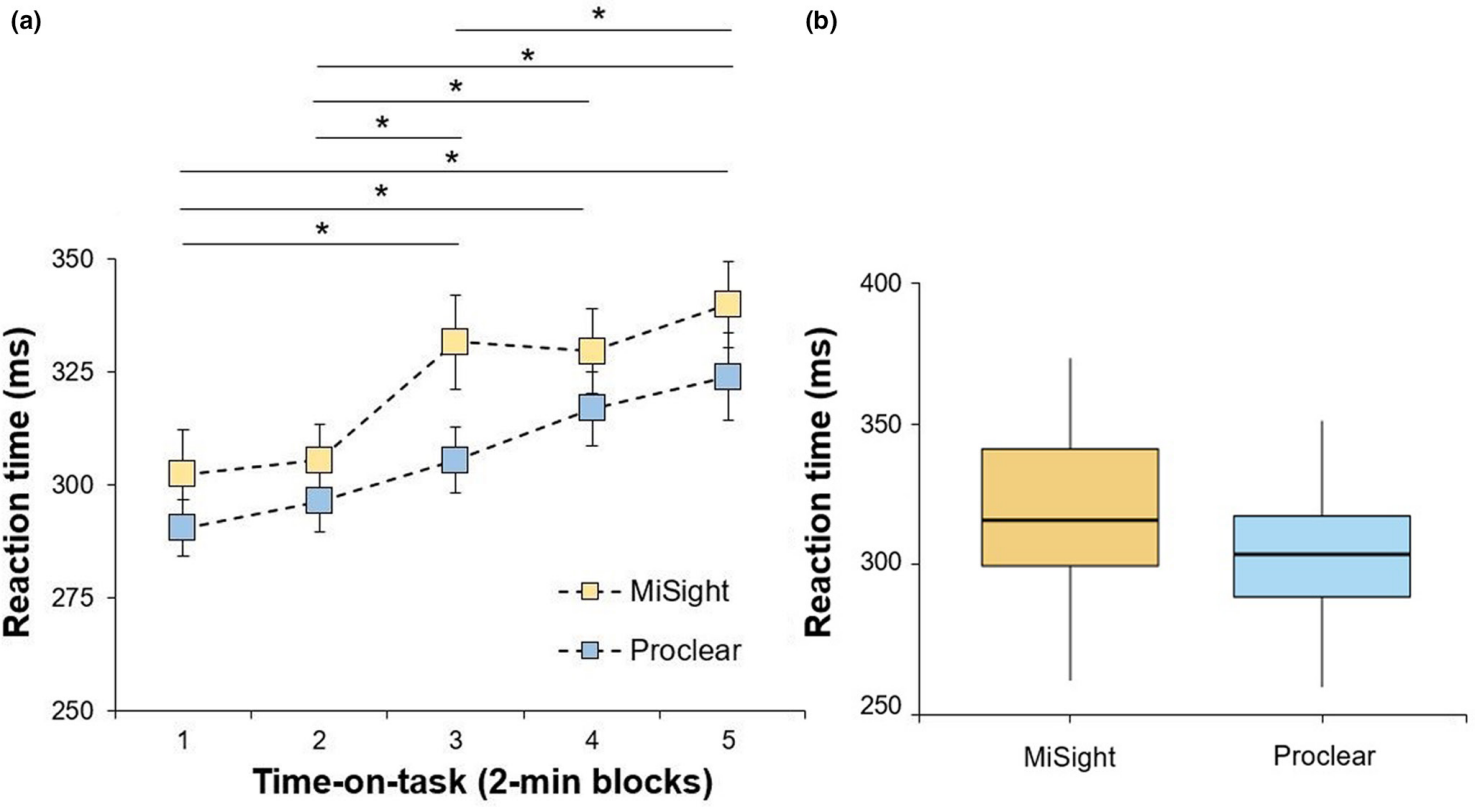
(a) Mean reaction time during the five time‐on‐task blocks for the MiSight and Proclear lenses. Error bars indicate one standard error. (b) Boxplot showing the mean variability with the whiskers extending 1.5 times the interquartile range from the 25th and *denotes statistically significant differences between the different time blocks (corrected *p*‐value < 0.05)

Finally, reaction time also showed statistically significant differences with lens type (MiSight: 321.86 ± 42.88 ms; Proclear: 306.70 ± 35.04 ms; *F*_1,23_ = 12.63, *p* = 0.002, ƞ_p_^2^ = 0.35), and the time‐on‐task (*F*_4,92_ = 29.88, *p* <0.001, ƞ_p_^2^ = 0.57), but not for the interaction lens type × time‐on‐task (*F*_4,92_ = 1.84, *p* = 0.13) (Figure 5). *Post‐hoc* comparisons are depicted in Figure 5a, and showed statistically significant differences for the block 1 vs. block 3 (corrected *p*‐value < 0.001, ES = 1.16), block 4 (corrected *p*‐value < 0.001, ES = 1.10) and block 5 (corrected *p*‐value < 0.001, ES = 1.86); block 2 vs. block 3 (corrected *p*‐value < 0.001, ES = 0.93), block 4 (corrected *p*‐value < 0.001, ES = 1.17) and block 5 (corrected *p*‐value < 0.001, ES = 1.62) and block 3 vs. block 5 (corrected *p*‐value = 0.004, ES = 0.70).

## DISCUSSION

The present study was designed to explore whether wearing dual‐focus soft contact lenses for myopia management alters the accommodative response and behavioural performance during the execution of a sustained near viewing task. The results showed that dual‐focus soft contact lenses increased both the lag and variability of accommodation and reaction time during a 10‐min PVT when compared with single‐vision soft contact lenses. Additionally, visual symptoms related to the clarity of vision were higher when wearing the dual‐focus lenses in comparison with the single‐vision lenses. These results confirm that accommodative function is altered by the use of dual‐focus lenses in the short term, and that these effects have a negative impact on behavioural performance.

The perceived levels of alertness/sleepiness (SSS) before each experimental session were assessed to control possible confounding factors. This measurement was not close to showing statistical significance, allowing us to confirm that participants attended each experimental session with similar alertness/sleepiness. While not the main objective of the current study, participants were asked to complete a 5‐point Likert scale with five questions to check their perceived level of visual discomfort after the 10‐min PVT. The results showed that dual‐focus soft contact lens wear impaired subjective clarity of vision; however, their use did not provoke tiredness in the eyes and head, eye strain or headache. These results agree with previous studies exploring visual symptomatology while wearing multifocal contact lenses at the fitting session and during a short follow‐up period.^23^, ^24^ Diec et al.^24^ reported poorer visual clarity amongst other symptoms (e.g., ghosting, vision stability, visual satisfaction and comfort) with multifocal in comparison with single‐vision contact lenses. Similarly, Kang and colleagues^23^ observed reduced quality of vision with multifocal contact lenses when evaluated with the Quality of Vision questionnaire. However, Huang et al.^25^ did not note differences in visual symptomatology between multifocal soft contact lenses and single‐vision spectacles based on the total score from the Quality of Vision questionnaire, which could be due to the longer follow‐up (1 month) and the neuroadaptation process occurring during this period.^41^

There is evidence that dual‐focus soft contact lenses increase the lag of accommodation in comparison to single‐vision lenses, possibly because observers use the positive zone to relax accommodation.^20^, ^34^, ^39^, ^42^ For example, previous reports found an increased lag of accommodation with multifocal soft contact lenses of 1.20 D at 40 cm in children [+2.50 D addition],^20^ and of 0.19 D and 0.49 D at 33 cm in adults (using +1.50 D and +3.00 additions).^22^ These results are in agreement with our findings as we also observed a significantly increased lag of accommodation at 50 cm with dual‐focus soft contact lenses (mean difference of 0.58 D). This could indicate the (partial) use of the positive zone of the MiSight lens during near vision to relax accommodation. The variations in accommodative lag across these studies may be explained by the different accommodative demands, visual stimuli and participants' ages. Although both studies^20^, ^22^ measured the accommodative lag objectively, they did not evaluate accommodation during the execution of an attentional demanding task at near.

Data from the present study revealed greater instability of accommodation with dual‐focus contact lenses while performing a 10‐min visual vigilance task. In this sense, Kajita and co‐workers^43^ demonstrated that accommodative stability is modulated as a function of the contact lens design. However, only Shibata et al.^44^ have compared the effects of wearing multifocal and single‐vision soft contact lenses on accommodative fluctuations, obtaining higher fluctuation values for the multifocal design. The increased variability of accommodation with the dual‐focus design could be explained by a conflict between the two dioptric powers of the lens, and associated changes in the perceived retinal image.^16^

Dual‐focus soft contact lenses have been shown to be an effective treatment to slow myopia progression by reducing relative peripheral hyperopia.^6^, ^11^, ^12^, ^14^ However, previous studies suggest that this optical design negatively affects visual performance, and could have a detrimental effect on real‐world contexts.^17^, ^45^ Some authors have shown that this optical design impairs visual acuity^20^, ^23^ and optical quality.^46^ However, there are also investigations that found these deficits recovered after an adaptation period.^6^, ^18^, ^24^ Regarding visual performance in applied settings, Gregory et al.^19^ compared binocular reading acuity and reading performance (words per minute) in multifocal and single‐vision contact lenses, noting that reading performance was worse with multifocal contact lenses. In the present study, we observed altered visual performance during the execution of a near task with the dual‐focus lenses; specifically, subjects had a slower reaction time while wearing the dual‐focus soft contact lenses (mean difference of 15.16 ms). This finding suggests that behavioural performance during sustained near vision is compromised with these lenses, and their use could affect other daily activities that require optimal visual performance (e.g., pilots, air traffic controllers, army personnel, surgeons, athletes, etc.).

The current findings may be of clinical relevance for the prescription of dual‐focus soft contact lenses for myopia management, and eye care practitioners should be aware of the impact of wearing dual‐focus soft contact lenses on accommodation and behavioural performance in order to prescribe the most beneficial optical solution according to the patient's visual needs. Nevertheless, this study has limitations and they should be acknowledged. First, all visual variables were evaluated after a short adaptation period (30 min) in order to examine the tolerance to these lenses at the first session since early discomfort may lead to low adherence and treatment compliance. However, there is evidence that multifocal contact lenses wearers experience neuroadaptive adaptation through recruitment of visual attentional and procedural learning networks.^41^ Indeed, previous studies reported an improvement in some visual parameters after periods of weeks or months.^23^, ^25^ Therefore, it is plausible that the variables measured here could improve after an adaptation period to this specific optical design. Further studies are needed in this regard. Second, the visual task chosen here consisted of a reaction time task. However, there are other visually‐ and cognitively‐demanding tasks that could be influenced by this optical design. It would be of interest to investigate further the effects of these lenses on different visual and mental tasks in applied scenarios (e.g., driving, sport, air traffic control, combat aircraft piloting, surgical procedures, etc.). Third, the accommodative and visual performance may be dependent on the refractive error, participant's age and optical design, and thus, our results cannot be extrapolated to other cohorts or lens types. Lastly, heightened ocular dryness or alterations in the blink pattern associated with the use of soft contact lenses may impact visual measurements, although it is plausible that these effects may be similar with both types of contact lens used in this study. We recommend that future studies should consider the inclusion of different refractive groups, children, clinical populations and other optical designs (e.g., multiple concentric treatment zones, spherical aberration) to determine the generalisability of the present results.

## CONCLUSION

The findings of the present study show that wearing dual‐focus soft contact lenses negatively affects accommodation (accommodative lag and variability) and behavioural performance (reaction time) during the execution of a 10‐min sustained visual task in the short‐term. These outcomes are of special relevance for practitioners when prescribing these lenses to slow myopia progression, especially in individuals who have demanding visual requirements. Future studies are needed to assess the long term‐effects of these lenses on accommodation and behavioural performance.

## AUTHOR CONTRIBUTIONS

**Beatriz Redondo:** Conceptualization (equal); formal analysis (equal); investigation (equal); methodology (equal); visualization (equal); writing – original draft (lead); writing – review and editing (equal). **Rubén Molina:** Data curation (equal); investigation (equal); methodology (equal); writing – review and editing (equal). **Tomás Galán:** Data curation (equal); investigation (equal); methodology (equal); writing – review and editing (equal). **Pedro Machado:** Data curation (equal); investigation (equal); methodology (equal); writing – review and editing (equal). **Raimundo Jiménez:** Conceptualization (equal); formal analysis (equal); investigation (equal); project administration (lead); supervision (lead); writing – review and editing (equal).
